# Cytoprotective Evaluation of *Juglans regia* L. Septum Extract and Development of Extract-Based Gels and Films for Topical Application

**DOI:** 10.3390/pharmaceutics18101329

**Published:** 2026-10-20

**Authors:** Jurgita Luksiene, Laura Rimkiene, Daiva Majiene, Jurga Andreja Kazlauskaite, Jurga Bernatoniene, Nerija Zevzikovaite, Andrejus Zevzikovas

**Affiliations:** 1 Department of Analytical and Toxicological Chemistry, Lithuanian University of Health Sciences, Sukileliu pr. 13, LT-50161 Kaunas, Lithuania; laura.rimkiene@lsmu.lt (L.R.); nerija.zevzikovaite@stud.lsmu.lt (N.Z.); andrejus.zevzikovas@lsmu.lt (A.Z.); 2 Department of Drug Technology and Social Pharmacy, Lithuanian University of Health Sciences, Sukileliu pr. 13, LT-50161 Kaunas, Lithuania; daiva.majiene@lsmu.lt (D.M.); jurga.andreja.kazlauskaite@lsmu.lt (J.A.K.); jurga.bernatoniene@lsmu.lt (J.B.); 3 Laboratory of Biochemistry, Neuroscience Institute, Lithuanian University of Health Sciences, LT-50162 Kaunas, Lithuania; 4 Institute of Pharmaceutical Technologies, Lithuanian University of Health Sciences, LT-50161 Kaunas, Lithuania

**Keywords:** *Juglans regia*, by-product, extract, fibroblasts, films, gel, three-dimensional printing

## Abstract

**Background/Objectives:** *Juglans regia* L. septa are a rich source of phenolic compounds with potential skin-related biological activity. This study evaluated the cytoprotective activity of a lyophilised *J. regia* septum extract and developed extract-containing polymeric gels suitable for topical application and semi-solid extrusion (SSE) 3D printing. **Methods:** The extract was characterised by high-performance liquid chromatography with photodiode array detection (HPLC-PDA) and evaluated in H_2_O_2_-exposed human fibroblasts. Poly(vinyl alcohol) (PVA)-, sodium carboxymethyl cellulose (CMC)-, and hydroxyethyl cellulose (HEC)-based gels were prepared, and the selected formulation was processed into films by solvent casting and SSE 3D printing. The films were evaluated for thickness, moisture content, mechanical properties, and probe–film adhesiveness. **Results:** Ellagic acid was the predominant quantified phenolic compound (36.453 µg/mL). The free extract protected fibroblasts against H_2_O_2_-induced oxidative damage, with the greatest preservation of viable cells at 75 and 100 µg/mL. Increasing extract loading increased total quantified phenolic content in the gels from 10.95 to 27.98 µg/mL. The selected formulation was successfully processed into multilayer films. 3D-printed films showed numerically lower burst strength and breaking force per unit width than solvent-cast films, although pairwise differences were not significant. Hydration increased probe–film adhesiveness, while film moisture content decreased during 3 months of storage. **Conclusions:** These findings support the feasibility of incorporating cytoprotective *J. regia* extract into printable polymeric gels for topical film development.

## 1. Introduction

Walnut (*Juglans regia* L.) septa have gained attention as a source of phenolic compounds with antioxidant, antimicrobial, and anti-inflammatory activities [1,2,3,4,5]. Their phytochemical profile includes hydrolysable tannins, ellagic acid and its derivatives, gallic acid, and flavonoids, although the relative abundance of these compounds depends on extraction conditions and geographical origin [1,2]. However, evidence from chemical antioxidant assays alone cannot establish whether these phytochemicals protect cells exposed to oxidative injury.

Fibroblasts play an important role in skin repair through extracellular matrix synthesis, collagen deposition, tissue remodelling, and wound contraction [6]. Oxidative stress strongly affects their function. While physiological levels of reactive oxygen species (ROS) contribute to cellular signalling and antimicrobial defence, excessive ROS can impair mitochondrial function, disrupt antioxidant defence mechanisms, and interfere with pathways involved in extracellular matrix maintenance [7,8]. Therefore, H_2_O_2_-exposed fibroblasts provide a useful in vitro model for evaluating whether antioxidant-rich extracts can preserve cell viability under oxidative-stress conditions. Previous studies have shown that polyphenol-rich plant extracts can reduce peroxide-induced cellular damage, while phenolic constituents associated with *J. regia*, including ellagic and gallic acids, have demonstrated cytoprotective effects in oxidative-stress models [9,10,11,12,13].

The therapeutic potential of a biologically active plant extract depends not only on its intrinsic activity but also on its successful incorporation into a delivery system capable of maintaining functionality at the wound site [14]. Hydrophilic polymeric systems are particularly relevant for this purpose because their high water affinity can provide conformability to the wound surface, support moisture retention and facilitate the local availability of incorporated active compounds [15,16,17]. For wound-oriented applications, however, a polymeric system’s performance depends on a balance of physicochemical and mechanical properties. An appropriate wound-contact material should maintain a sufficiently moist environment while avoiding excessive fluid accumulation, permit gaseous exchange, conform to the application surface, and retain sufficient mechanical integrity during handling and use [18,19]. Adhesive properties must likewise provide adequate contact with the application surface without causing additional tissue damage during removal [20]. These requirements make hydration, residual moisture, adhesion and mechanical behaviour functionally relevant attributes rather than solely technological characteristics of the formulation.

The properties of such gel–film systems are governed by the composition and intermolecular organisation of the polymeric network. Polymer type and concentration, plasticiser content, and residual water influence chain mobility, viscosity, hydration, and mechanical behaviour, while incorporating complex botanical extracts introduces additional interactions that may modify these characteristics [21,22]. This is particularly relevant to formulations based on cellulose derivatives and poly(vinyl alcohol) (PVA). Maslii et al. demonstrated pronounced formulation-dependent differences in thickness, moisture content, bursting strength, tensile behaviour and adhesion in plant-active-containing films prepared using sodium carboxymethylcellulose (CMC), hydroxyethyl cellulose (HEC), PVA and plasticisers including polyethylene glycol 400 (PEG 400) [23]. Consequently, formulation selection cannot rely solely on the mechanical strength of the dried film. The properties of the precursor gel and its subsequent transformation during drying must also be considered [24]. Preservation of the incorporated phytochemicals must also be considered during this transformation. Phenolic compounds may be susceptible to degradation as a function of temperature, oxygen exposure, light and pH, making their retention during processing and subsequent storage an important quality attribute of extract-loaded polymeric systems [25,26].

Semi-solid extrusion (SSE) 3D printing provides a means of converting a polymeric gel directly into a structured film through layer-by-layer deposition. Its success depends strongly on the properties of the precursor formulation, particularly its consistency, homogeneity, extrudability, and ability to retain shape after deposition [27,28,29]. Previous studies have separately demonstrated that plant-derived extracts can be incorporated into SSE-printable matrices [30,31,32,33]. Additive manufacturing enables control over film dimensions, internal architecture, and multifunctional design [33,34,35], while processing and drying conditions can substantially influence the final properties of polymeric films [36]. However, researchers have generally investigated these aspects independently rather than within a single integrated extract–gel–film system.

Despite these advances, the incorporation of *J. regia* septum extract into a polymeric gel–film system suitable for SSE 3D printing has not been systematically investigated. In particular, information is lacking on how incorporating the extract affects precursor-gel properties, printability, phenolic composition, and the physicochemical and mechanical characteristics of the resulting films, and how these properties compare with conventionally cast films. Comparison with conventional casting is therefore necessary to distinguish formulation-dependent effects from those introduced by the additive manufacturing process and to establish whether the biologically active extract can be translated into a technologically robust wound-oriented delivery system. Therefore, this study aimed to evaluate the biological activity of lyophilised *Juglans regia* L. septum extract and subsequently develop extract-loaded polymeric films, assessing their suitability for semi-solid extrusion 3D printing, physicochemical and mechanical properties, chemical composition, and stability compared with conventionally cast films.

## 2. Materials and Methods

### 2.1. Materials and Reagents

Poly(vinyl alcohol) (PVA; 87–90% hydrolysed, average molecular weight 30,000–70,000; product no. P8136), hydroxyethyl cellulose (HEC; average molecular weight 380,000), sodium carboxymethyl cellulose (CMC; average molecular weight 250,000), polyethylene glycol 400 (PEG 400), and propylene glycol were purchased from Sigma-Aldrich (St. Louis, MO, USA). Ethanol (96%) was purchased from Vilniaus degtinė (Vilnius, Lithuania). D-panthenol (75%), sodium hyaluronate (medium molecular weight), and glycerin (98–100%, palm oil-free, organic) were supplied by alexmo cosmetics GmbH (Stuhr, Germany). Purified water complying with the requirements of European Pharmacopoeia monograph 0008 was produced in the LSMU laboratory (Kaunas, Lithuania) using a Milli-Q water purification system (Merck KGaA, Darmstadt, Germany). Acetonitrile (high-performance liquid chromatography grade, 99.9%) was obtained from Fisher Scientific (Waltham, MA, USA), while trifluoroacetic acid (HPLC grade, ≥99.0%) was purchased from Sigma-Aldrich (St. Louis, MO, USA). Pedunculagin/casuarinin isomers I and II, ellagic acid pentoside, and caffeoyl hexose-deoxyhexoside were obtained from Sigma-Aldrich (St. Louis, MO, USA).

Human BJ-5ta fibroblast cells (ATCC CRL-4001, RRID: CVCL_6573, American Type Culture Collection, Manassas, VA, USA) were used in the study. Dulbecco’s Modified Eagle Medium (DMEM), penicillin–streptomycin solution, trypsin–EDTA solution, and hydrogen peroxide (H_2_O_2_) were purchased from Sigma-Aldrich Chemie GmbH (Steinheim, Germany). Fetal bovine serum (FBS) was obtained from Gibco (Thermo Fisher Scientific, Waltham, MA, USA). Hoechst 33342 and propidium iodide were purchased from Sigma-Aldrich (Steinheim, Germany).

### 2.2. Plant Origin and Extract Preparation

*Juglans regia* L. fruits were collected in autumn 2024 (late September–early October) in Šiauliai, Lithuania (56.07331, 23.46924), from two mature, ungrafted walnut trees growing in a homestead environment. Approximately 2 kg of fully mature fruits were collected after natural abscission. The fruits were dried at room temperature for 2–3 weeks, after which the shells were opened and the septa were manually separated. The septa were milled and stored in glass containers in a dark, dry place at room temperature until extraction.

The plant extract was prepared from *J. regia* plant material using ethanolic extraction followed by solvent removal and lyophilisation. Briefly, 4 ± 0.01 g of plant raw material was mixed with 80 mL of 60% (*v*/*v*) ethanol. The mixture was heated under reflux at 90 °C for 1 h. After extraction, the obtained mixture was removed from the heat source, allowed to cool to room temperature, and filtered through paper filters to separate the liquid extract from the solid plant residue.

The filtrate was concentrated using a rotary evaporator (Heidolph Instruments GmbH & Co. KG, Schwabach, Germany) until approximately 40% of the initial liquid volume remained. This step removed the ethanol fraction from the extract solution. The resulting aqueous extract concentrate was then frozen and lyophilised using a freeze dryer (Christ Gamma 1–15 LSC, Martin Christ Gefriertrocknungsanlagen GmbH, Osterode am Harz, Germany). The obtained dry extract was collected and used for further experimental studies. The extraction procedure yielded approximately 0.30 g of lyophilised extract from 4.0 g of plant material, corresponding to an extraction yield of 7.5% (*w*/*w*), calculated relative to the initial dry plant material.

### 2.3. Cultivation of Fibroblast Cells

Fibroblast cells were maintained under standard in vitro culture conditions. The cells were grown in 75 cm^2^ culture flasks containing 9 mL of complete growth medium. The medium was composed of DMEM supplemented with 10% fetal bovine serum and 1% penicillin–streptomycin solution. Cultures were incubated at 37 °C in a humidified atmosphere containing 5% CO_2_.

The growth medium was renewed every 2–3 days to provide sufficient nutrients and remove metabolic waste products. Cells were used for further experiments when the monolayer reached approximately 80–90% confluence. At this stage, cells were detached and reseeded for the planned experimental procedures.

The cells were counted and seeded into well plates at a density of 20,000 cells per well. After seeding, the plates were incubated for 24 h to allow the cells to attach before exposure to oxidative stress and extract treatment.

### 2.4. Oxidative Stress Model and Treatment with Juglans regia L. Extract

An oxidative stress model was established by treating fibroblast cells with hydrogen peroxide. After 24 h of cell attachment, 800 µM H_2_O_2_ was added to the experimental wells. At the same time, the cells were exposed to different concentrations of *J. regia* extract.

The extract was tested at concentrations of 25, 50, 75, and 100 µg/mL. After the addition of H_2_O_2_ and the different concentrations of the extract, the cells were incubated for 24 h under standard culture conditions.

Untreated fibroblasts were used as the control group. Cells treated only with 800 µM H_2_O_2_ were used as the oxidative stress control. Additional antioxidant comparison groups included cells treated with catalase under oxidative stress conditions.

### 2.5. Assessment of Fibroblast Viability

Cell viability after treatment was evaluated using fluorescence staining with Hoechst 33342 and propidium iodide (PI). Hoechst 33342 was applied at 15 µg/mL to visualise cell nuclei, while PI was applied at 5 µg/mL to identify cells with compromised plasma membranes.

**Figure 1:**
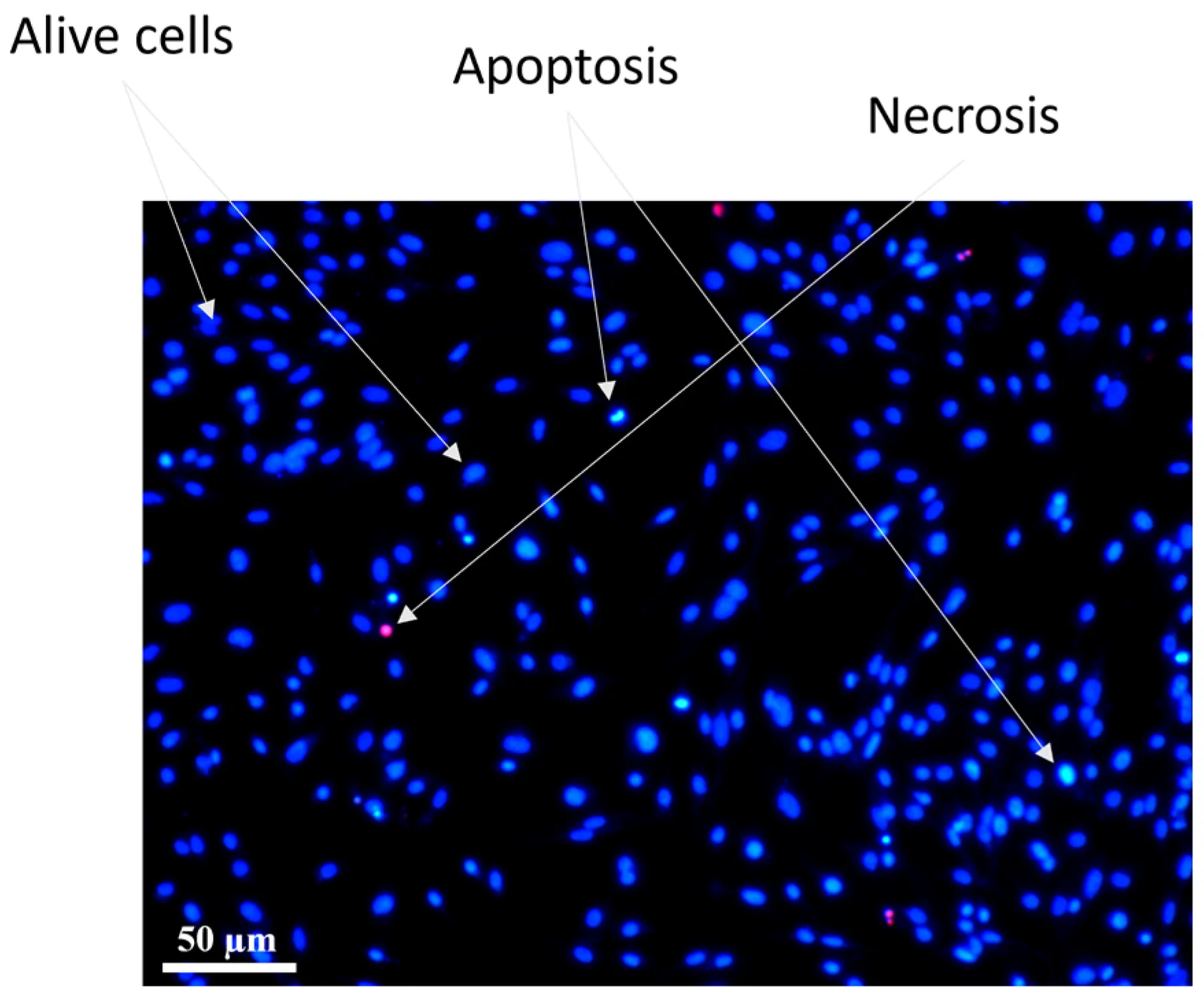
Representative fluorescence microscopy images illustrating the morphological classification of viable, apoptotic-like, and membrane-damaged/necrotic-like fibroblasts.

After staining, cells were examined using a fluorescence microscope. Three independent biological experiments were performed, with two wells per treatment in each experiment and at least five randomly selected microscopic fields analysed per well. Cells showing blue nuclear fluorescence with intact nuclear morphology and without PI uptake were considered viable. PI-positive cells were classified as membrane-damaged/necrotic-like. Small, intensely Hoechst-positive nuclei with condensed or fragmented chromatin were classified as apoptotic-like. Nuclei showing both Hoechst and PI fluorescence were considered membrane-compromised/non-viable and were not interpreted as evidence of a specific cell-death mechanism (Figure 1).

The obtained microscopic images were used to compare the effect of different *Juglans regia* L. extract concentrations on fibroblast viability under H_2_O_2_-induced oxidative stress. The morphological classification was used descriptively and was not intended to establish the molecular mechanism of cell death.

### 2.6. Gels Preparation

Several formulations were initially developed to obtain a gel suitable both for direct application and for subsequent 3D printing into films. The formulations had to provide sufficient viscosity for printing while remaining suitable for use as gels and capable of drying efficiently to form films. Based on these criteria, the formulations presented in Table 1 were selected as the final gel bases.

Formulation GIII was carried forward to subsequent studies and is hereafter referred to as G. A control gel without extract was prepared alongside three extract-loaded formulations containing 0.092, 0.138, and 0.184% (*w*/*w*) lyophilised extract. To maintain the same final formulation mass, the amount of purified water was reduced according to the amount of extract added.

The G formulation was prepared by dispersing PVA in purified water and heating the mixture to 80 °C under continuous stirring until complete dissolution. CMC and HEC were hydrated separately in purified water and subsequently combined with the PVA solution to obtain the polymeric gel base. PEG 400, propylene glycol, sodium hyaluronate, D-panthenol, and ethanol were mixed separately and incorporated into the gel base after cooling to approximately 50 °C. The lyophilised walnut septa extract was then added to the relevant formulations and mixed until a homogeneous gel was obtained (Table 2). The formulation prepared without the extract served as the control.

The extract loadings of 0.092, 0.138, and 0.184% (*w*/*w*) corresponded to 0.92, 1.38, and 1.84 mg/g of gel, respectively. These formulation loadings are not directly comparable to the free-extract concentrations used in the fibroblast experiments because this study did not evaluate extract release from the polymeric matrix. After preparation, the gels were transferred to 50 mL screw-cap tubes and the gels were stored at room temperature at a relative humidity of approximately 40–50% in tightly closed 50 mL screw-cap tubes under ambient laboratory light conditions. Gel pH and apparent viscosity were evaluated 24 h after preparation and after 3 months of storage.

### 2.7. Film Formation from Gels

An in-house semi-solid extrusion (SSE) 3D printer was used for film fabrication. The film-forming formulation was loaded into a 60 mL Omnifix^®^ syringe (B. Braun Melsungen AG, Melsungen, Germany and extruded through n 18 G stainless-steel needle tip with a diameter of 0.84 mm and a length of 31.5 mm. The printing model was prepared and sliced using PrusaSlicer software, version 2.9.2 (Prusa Research a.s., Czech Republic), using the SSE-LSMU printer profile and the SSE-60 mL BRAUN Omnifix material profile.

Rectangular films measuring 40 mm × 80 mm were printed using a rectilinear filling pattern in 10 layers with a programmed layer height of 0.20 mm. The print speed defined in PrusaSlicer was 25 mm/s. During printing, the printer speed override was set to 80% of the slicer-defined value, corresponding to a nominal effective printing speed of approximately 20 mm/s. The extrusion flow multiplier was set to 100%, indicating that no additional increase or decrease in the slicer-defined extrusion flow was applied.

A 180 s interlayer pause was introduced after each of the first nine layers to allow partial drying and stabilisation of the deposited material before deposition of the subsequent layer. The PrusaSlicer/G-code estimate of 14 min 45 s represented the active printing time calculated from the printing movements and did not include the programmed waiting periods. Therefore, the nine interlayer pauses added 27 min, resulting in an approximate total process time of 41 min 45 s. Approximately 6.9 g of formulation was used for each film, and the printing bed was maintained at 40 °C throughout the printing process.

For comparison, films were also prepared using the conventional solvent-casting method. The same amount of gel formulation, 6.9 g, was transferred into 40 × 80 mm moulds. The cast films were dried at 40 °C for 42 min, after which they were subsequently kept at room temperature until 48 h after preparation. After printing, the 3D-printed films were kept at room temperature for 48 h before further analysis.

Gel formulations were labelled with G, and the corresponding films were labelled with F, while keeping the same number. Films produced by 3D printing were additionally marked with D. Films prepared by the conventional solvent-casting method were used as reference samples and therefore had no additional letter. For example, G1 represents the gel, F1 the solvent-cast film, and F1D the corresponding 3D-printed film.

For the 3-month stability evaluation, the films were stored at room temperature at a relative humidity of approximately 40–50% in transparent packaging under ambient laboratory light conditions.

### 2.8. Analysis of the Products

#### 2.8.1. Apparent Viscosity

Viscosity was determined using a Fungilab ALPHA series viscometer (Fungilab, Sant Feliu de Llobregat, Barcelona, Spain). For each measurement, 50 mL of sample was transferred into the viscometer vessel and placed on the instrument platform. Measurements were carried out at room temperature using an L2 spindle at 100 rpm. Apparent Viscosity was recorded in mPa·s once the reading had stabilised. The analysis was repeated five times, and the average value was calculated. Measurements were performed after 24 h of gel preparation and after 3 months of storage.

As measurements were performed at a single rotational speed, the obtained values represent apparent viscosity under the specified measurement conditions and do not constitute comprehensive rheological characterisation. Printability was additionally assessed qualitatively during SSE based on continuous material flow, absence of nozzle clogging, and retention of the deposited structure.

#### 2.8.2. pH Measurement

Gel pH was evaluated with a Thermo Scientific Orion VERSA STAR Advanced Electrochemistry Meter (Beverly, MA, USA). During analysis, the electrode was introduced directly into the formulation and the displayed value was recorded only after it had become constant. Five measurements were obtained for each sample, and the average pH was calculated. Measurements were taken after 24 h of gel preparation and after 3 months of storage.

#### 2.8.3. Thickness Measurement of the Films

Film thickness was determined using an Electronic Digital Caliper DIN 862 (Vogel Germany GmbH & Co. KG, Kevelaer, Germany). Each film was measured at five different points, and the mean thickness value was calculated. For each formulation, three individual films were analysed. The film measurements were done after 48 h of preparation and after 3 months of storage.

#### 2.8.4. Moisture Measurement

Moisture content was determined using a KERN DBS 60-3 moisture analyser (KERN & SOHN GmbH, Balingen, Germany). Approximately 0.10 ± 0.05 g of each sample was placed in the analyser and dried at 105 °C until no further change in mass was observed. The moisture percentage was calculated automatically from the reduction in sample mass during drying. Film samples were analysed 48 h after preparation and again after 3 months of storage. Each formulation was tested in triplicate.

#### 2.8.5. Texture Analysis of Films

##### Tensile Breaking Test

The analysed films were cut to 6 × 1 cm in size and attached at both ends to clamps on opposite sides of the A/TG probe. The clamps were held on a metal base, with one clamp fixed and the other movable. During measurement, the movable clamp pulled the film upward at a speed of 6 mm/s, with a maximum lifting distance of 150 mm. The force at film rupture was recorded and expressed as breaking force per unit width (N/mm). Because all specimens had a width of 10 mm, the measured breaking force was normalized to specimen width. Five independent film specimens were analysed for each formulation at room temperature.

##### Assessment of Adhesion

A completely dry film sample measuring 2 × 2 cm was placed on a fixed platform and secured with a transparent plastic plate containing a 1 cm diameter circular opening. For hydrated samples, the exposed film surface was moistened with 35 µL of water and allowed to hydrate for 15 s. A P/0.5R flat-ended probe was pressed onto the film surface with a contact force of 5 N for 60 s. The probe was then withdrawn, and the maximum force required to detach the probe from the film surface was recorded as probe–film adhesiveness (N). The work of adhesion (N·s) was determined as the area under the force–time curve during probe withdrawal. Five replicate measurements were performed for each sample at room temperature. These measurements characterise adhesion at the probe–film interface under the specified experimental conditions and should not be interpreted as direct measurements of adhesion to human skin.

### 2.9. HPLC-PDA Analysis of the Lyophilised Extract and Gels

Extract samples were prepared according to the methodology described in our previous study [5]. Extract-loaded gel samples were diluted with 70% aqueous methanol at a sample-to-solvent ratio of 1:10 (*v*/*v*) prior to HPLC-PDA analysis.

HPLC-PDA analysis was carried out using a Waters e2695 separation module coupled with a Waters 2998 photodiode array (PDA) detector (Waters Corporation, Milford, MA, USA). Chromatographic separation was performed on an ACE C18 column (250 mm × 4.6 mm, 5.0 µm). The mobile phase consisted of solvent A (0.1% trifluoroacetic acid in water) and solvent B (acetonitrile), delivered at a constant flow rate of 1.0 mL/min [5].

Gradient elution was applied as follows: 95% A/5% B (0 min), 85% A/15% B (8 min), 80% A/20% B (30 min), 50% A/50% B (58–65 min), 5% A/95% B (66–70 min), followed by re-equilibration to initial conditions at 71 min. The column temperature was maintained at 25 °C, the injection volume was 10 µL, and detection was performed in the wavelength range of 200–400 nm [5].

Phenolic compounds were tentatively identified based on retention times and UV–Vis spectra obtained from PDA detection and by comparison with retention times of reference standards analysed under the same conditions. The HPLC-PDA method and its analytical performance were previously evaluated for *Juglans regia* L. septa extracts [5]. The reported limit of detection (LOD) and limit of quantification (LOQ) were established for ellagic acid and for quercitrin. Compound-specific LOD and LOQ values were not established for the remaining phenolic constituents. Therefore, the experimentally determined ellagic acid sensitivity parameters were used only as reference thresholds when evaluating low-level chromatographic responses of these compounds and were not considered analyte-specific LOD or LOQ values. Compounds producing identifiable chromatographic responses that could not be reliably quantified were therefore designated as not quantified (NQ).

### 2.10. Data Processing and Statistical Analysis

Fibroblast viability was evaluated in three independent experiments, with two wells per treatment and at least five randomly selected microscopic fields analysed per well. Results are presented as absolute cell counts, as H_2_O_2_ markedly reduced the overall cell population, and are expressed as mean ± SD.

Data are presented as mean ± standard deviation (SD). Statistical analysis was performed separately for each investigated parameter using one-way analysis of variance (ANOVA), followed by Tukey’s multiple-comparisons post hoc test. Structural-mechanical measurements were performed in quintuplicate (*n* = 5), and film burst strength and breaking force per unit width were analysed separately. A value of *p* < 0.05 was considered statistically significant.

## 3. Results

### 3.1. Determined Compounds in Juglans regia L. Extract

The chromatographic analysis of the lyophilised walnut septa extract resulted in the quantification of six phenolic compounds. The phytochemical profile was dominated by ellagic acid and its derivative, as ellagic acid showed the highest concentration (36.453 µg/mL), followed by ellagic acid pentoside (24.144 µg/mL) (Table 3). Together, these two compounds accounted for more than half of the quantified compounds, indicating that ellagic acid-related constituents were the predominant compounds in the extract.

Quercitrin was also detected at a relatively high concentration (25.501 µg/mL), confirming the presence of flavonoid glycosides in the extract. In addition, two pedunculagin/casuarin isomers were quantified at 13.891 and 15.098 µg/mL, respectively, showing the contribution of hydrolysable tannins to the overall phytochemical composition. Caffeoyl hexose-deoxyhexoside was present at the lowest concentration (4.95 µg/mL), but its detection indicates that caffeic acid derivatives were also present in the extract (Table 3).

Overall, the obtained phytochemical profile demonstrates that the lyophilised *Juglans regia* L. extract contains a complex mixture of phenolic compounds, which may contribute to its antioxidant and biological activity.

### 3.2. Protective Effect of Juglans regia L. Extract on Fibroblasts Under H_2_O_2_-Induced Oxidative Stress

**Figure 2:**
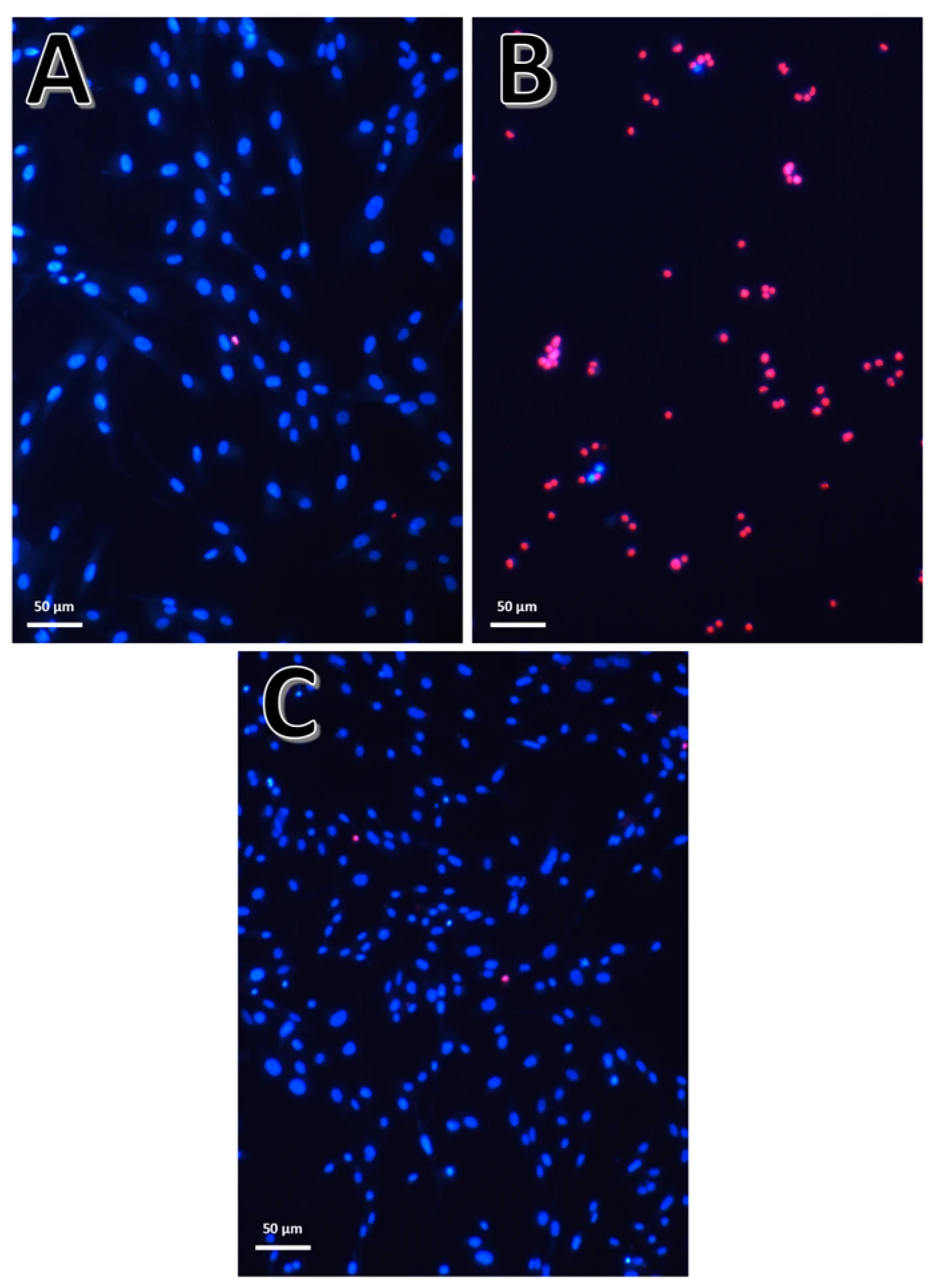
Images of fibroblasts under control and reference treatment conditions: (**A**) untreated control; (**B**) fibroblasts exposed to 800 µM H_2_O_2_; (**C**) fibroblasts treated with catalase under oxidative-stress conditions.

Exposure of fibroblasts to 800 µM H_2_O_2_ for 24 h resulted in a pronounced deterioration of the cell population compared with the untreated control. The untreated control was characterised predominantly by viable cells, whereas fibroblasts exposed to oxidative stress showed extensive cell damage and substantially fewer viable cells. Representative images clearly demonstrate the difference between the normal fibroblast culture and the H_2_O_2_-treated group (Figure 2A,B).

Catalase showed a clear protective effect against H_2_O_2_-induced damage (Figure 2C). The catalase-treated cultures contained considerably more viable fibroblasts than the oxidative-stress control and showed an overall cellular appearance closer to that of the untreated control.

**Figure 3:**
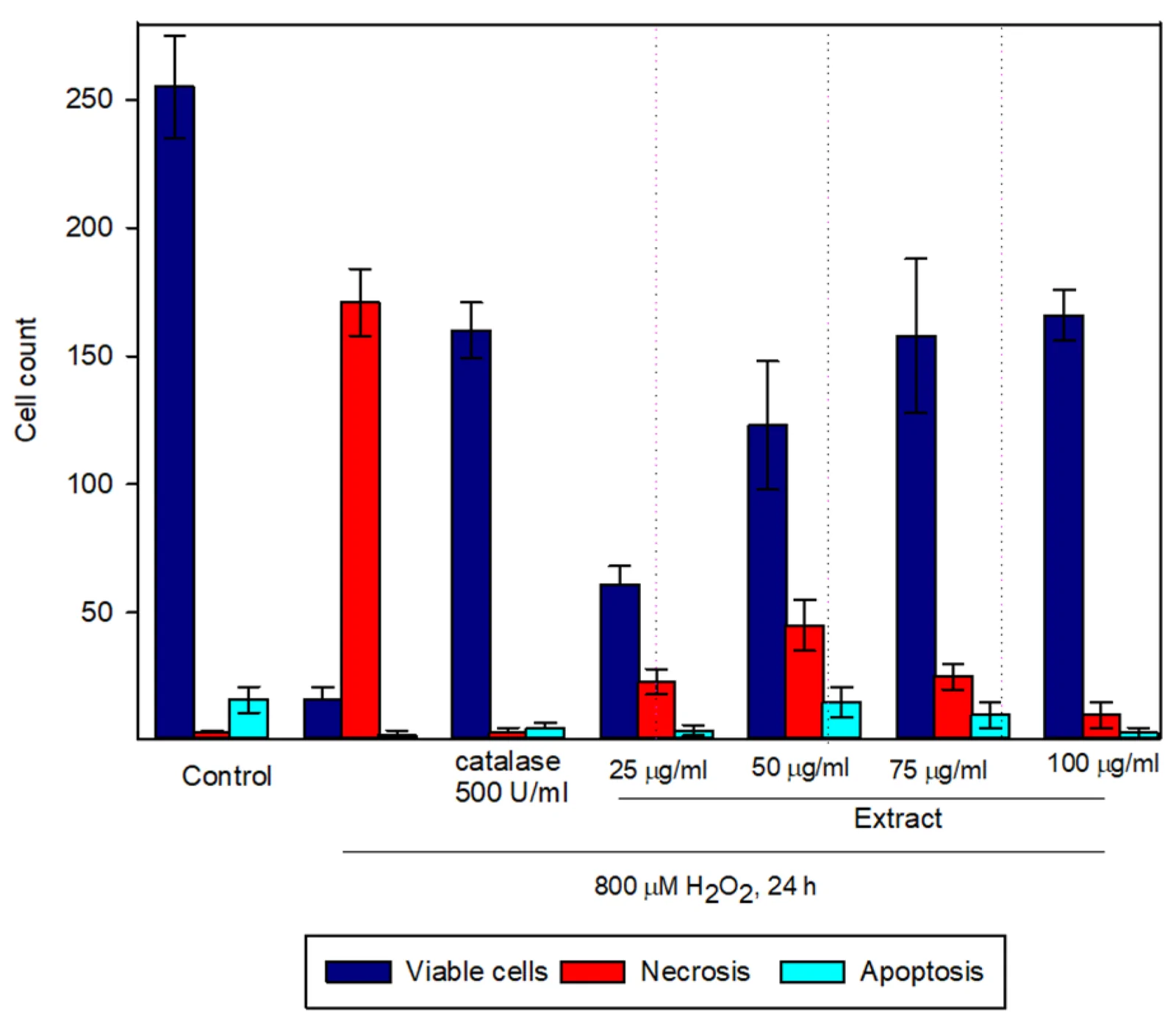
Quantitative comparison of viable, necrotic-like, and apoptotic-like fibroblasts in the untreated control and following exposure to 800 µM H_2_O_2_ for 24 h, with catalase (500 U/mL) or *Juglans regia* extract at 25, 50, 75, and 100 µg/mL. The oxidative-stress control received H_2_O_2_ without protective treatment. Data are presented as mean ± standard deviation (SD) from three independent experiments, with two wells per treatment and at least five randomly selected microscopic fields analysed per well. *p* < 0.05 was considered statistically significant.

Treatment with *J. regia* extract protected fibroblasts from oxidative-stress-induced damage, although the magnitude of the effect varied according to extract concentration (Figure 3). At 25 µg/mL, only a partial preservation of the fibroblast population was observed. Increasing the extract concentration to 50 µg/mL resulted in a visibly greater number of viable cells. The most pronounced protective response was observed at 75 and 100 µg/mL, at which the fibroblast populations were considerably better preserved than at the lower extract concentrations (Figure 3).

**Figure 4:**
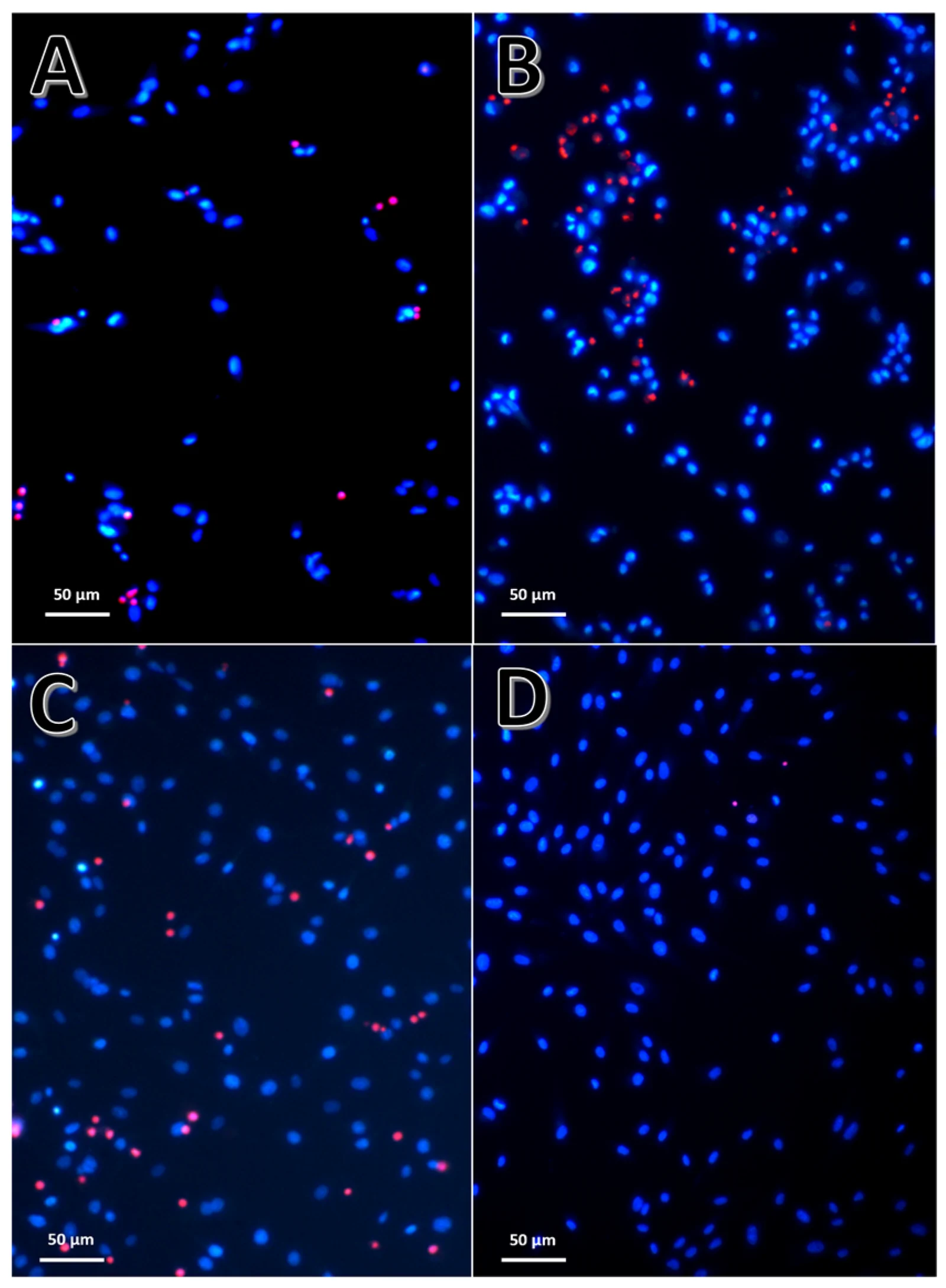
Representative images of fibroblasts treated with *Juglans regia* extract under oxidative-stress conditions at (**A**) 25 µg/mL, (**B**) 50 µg/mL, (**C**) 75 µg/mL, and (**D**) 100 µg/mL.

The untreated control showed the highest number of viable cells, approximately 255, with relatively few necrotic-like or apoptotic-like cells (Figure 3). Under oxidative-stress conditions, the viable cell population decreased markedly, whereas cell death increased substantially. Catalase considerably preserved the viable cell population, with viable cell numbers reaching approximately 170. The highest viable cell count among the extract-treated groups was observed at 100 µg/mL. Viable cell numbers at 75 and 100 µg/mL approached those observed in the catalase-treated group, indicating that the protective effect was greatest at the higher extract concentrations (Figure 3 and Figure 4).

The pattern of cell death also differed between the extract concentrations. The 25 µg/mL group showed comparatively high numbers of necrotic-like cells and the lowest preservation of viable fibroblasts among the extract-treated groups. At 50 µg/mL, the number of viable cells increased substantially, although necrotic-like cells remained evident. Further improvement was observed at 75 µg/mL, while treatment with 100 µg/mL produced the most favourable overall distribution, characterised by a high viable cell count and relatively low numbers of necrotic-like and apoptotic-like cells (Figure 4).

Overall, the quantitative and visual results consistently demonstrated a concentration-related protective effect of *J. regia* extract against oxidative-stress-induced fibroblast damage (Figure 4). The protective response increased particularly between 25 and 75 µg/mL, while the results obtained at 75 and 100 µg/mL were relatively similar. These two concentrations provided the greatest preservation of viable fibroblasts and produced responses approaching that observed with catalase.

### 3.3. Development and Physicochemical Characterization of the Gels

**Figure 5:**
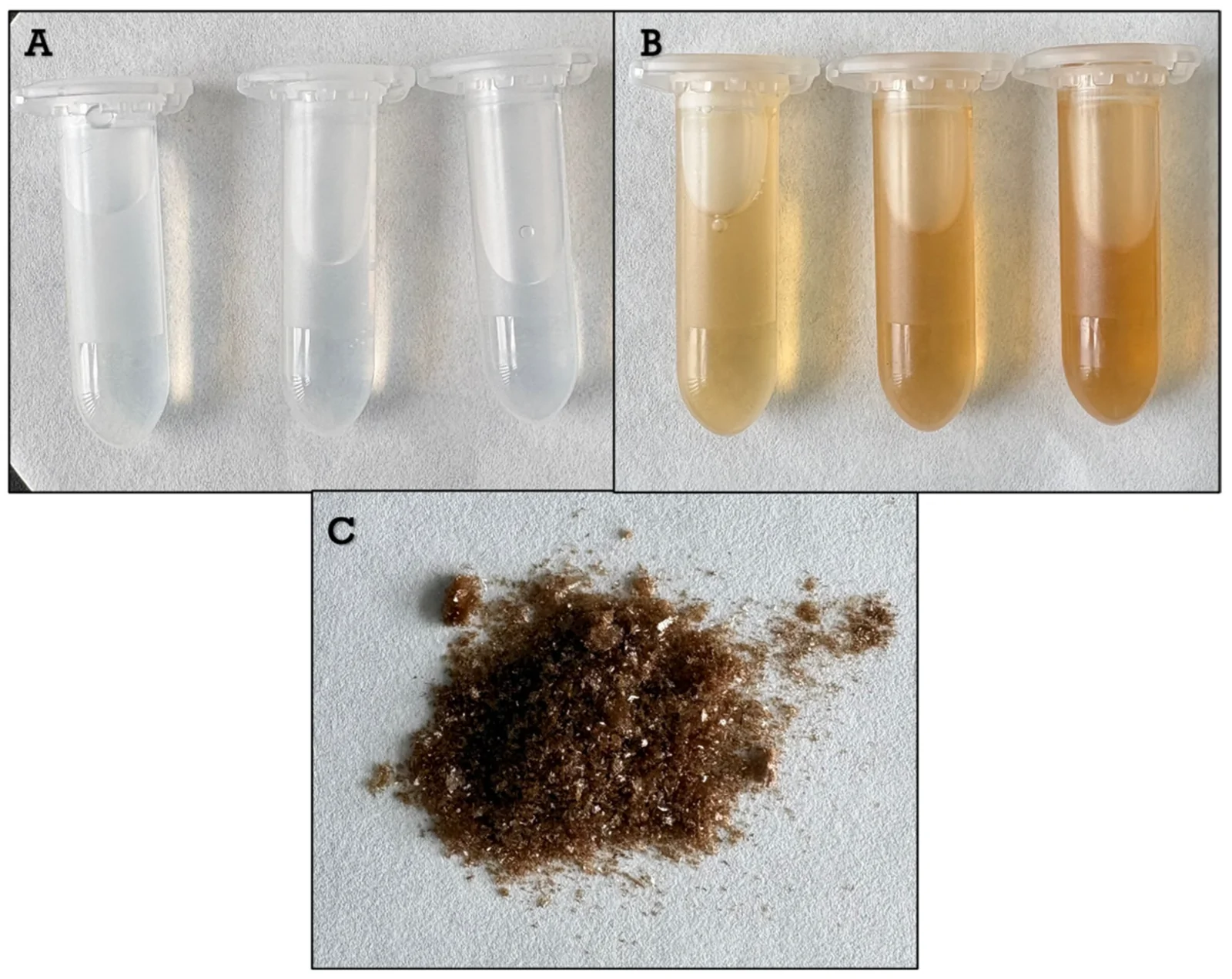
Images of (**A**) control gel formulations, from left to right: GI, GII, and G; (**B**) extract-containing formulations, from left to right: G1, G2, and G3; and (**C**) the lyophilised extract used for gel preparation.

**Figure 6:**
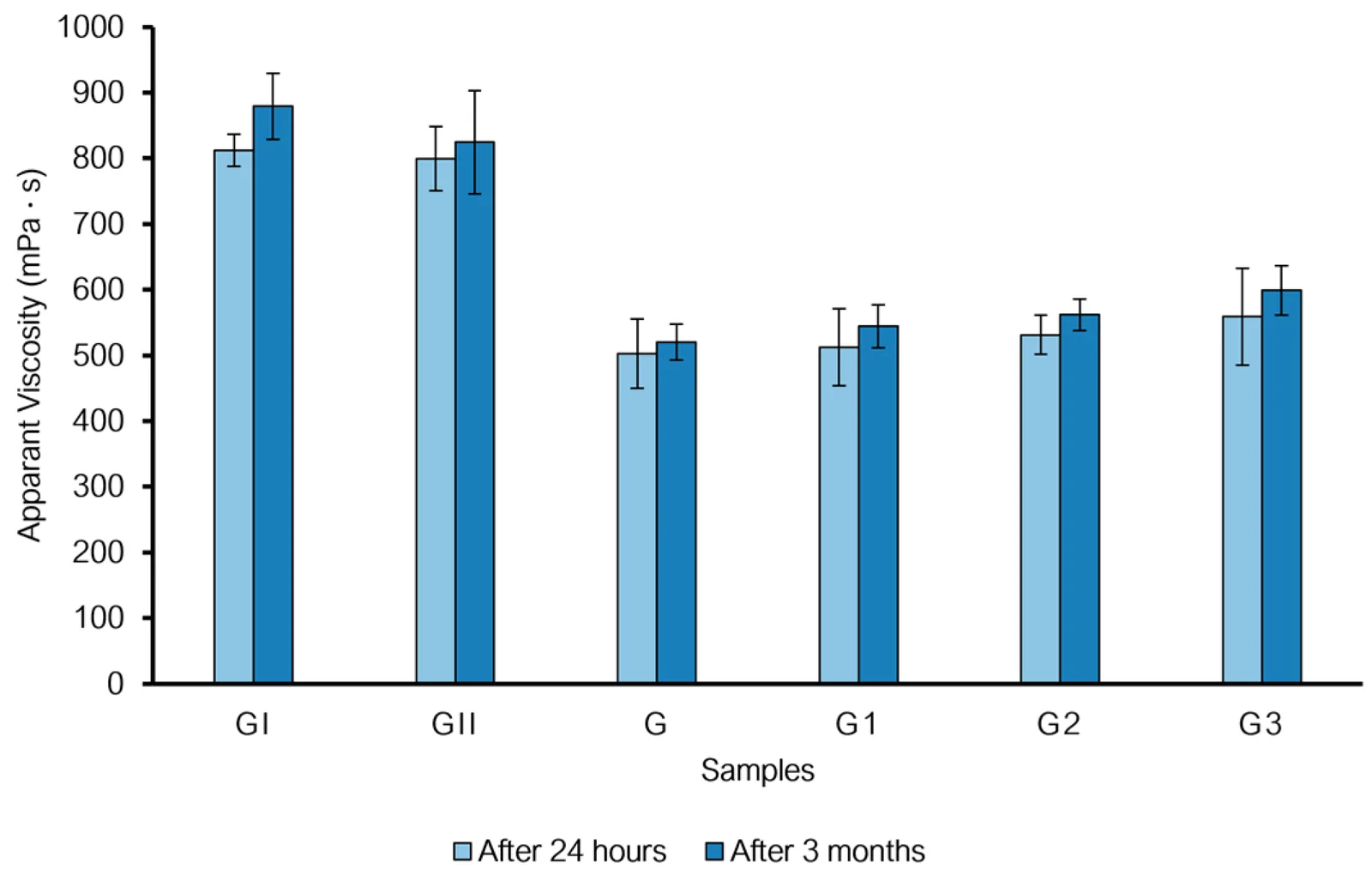
The apparent viscosity of the gels after 24 h and after 3 months (*n* = 5).

The investigated gel formulations (Figure 5A,B) differed in their apparent viscosity, with GI and GII showing higher values than formulation G (Figure 6). Formulation G also appeared homogeneous, with no visible dispersed particles. Based on these characteristics, formulation G was selected for incorporation of the lyophilised *J. regia* extract and for subsequent SSE 3D printing.

The addition of the lyophilised extract (Figure 5C) at 0.092% (G1), 0.138% (G2), and 0.184% (*w*/*w*) (G3) did not significantly change the consistency of the selected gel base compared with the extract-free control G. Although a slight numerical increase was observed with increasing extract concentration, the differences between G and the extract-containing formulations were not statistically significant. Furthermore, no significant changes in consistency were observed for any of the formulations after 3 months of storage compared with the initial measurements (*p* > 0.05). These findings indicate that incorporation of the extract did not adversely affect the consistency of the gel and that this physical property remained stable throughout the investigated storage period.

**Figure 7:**
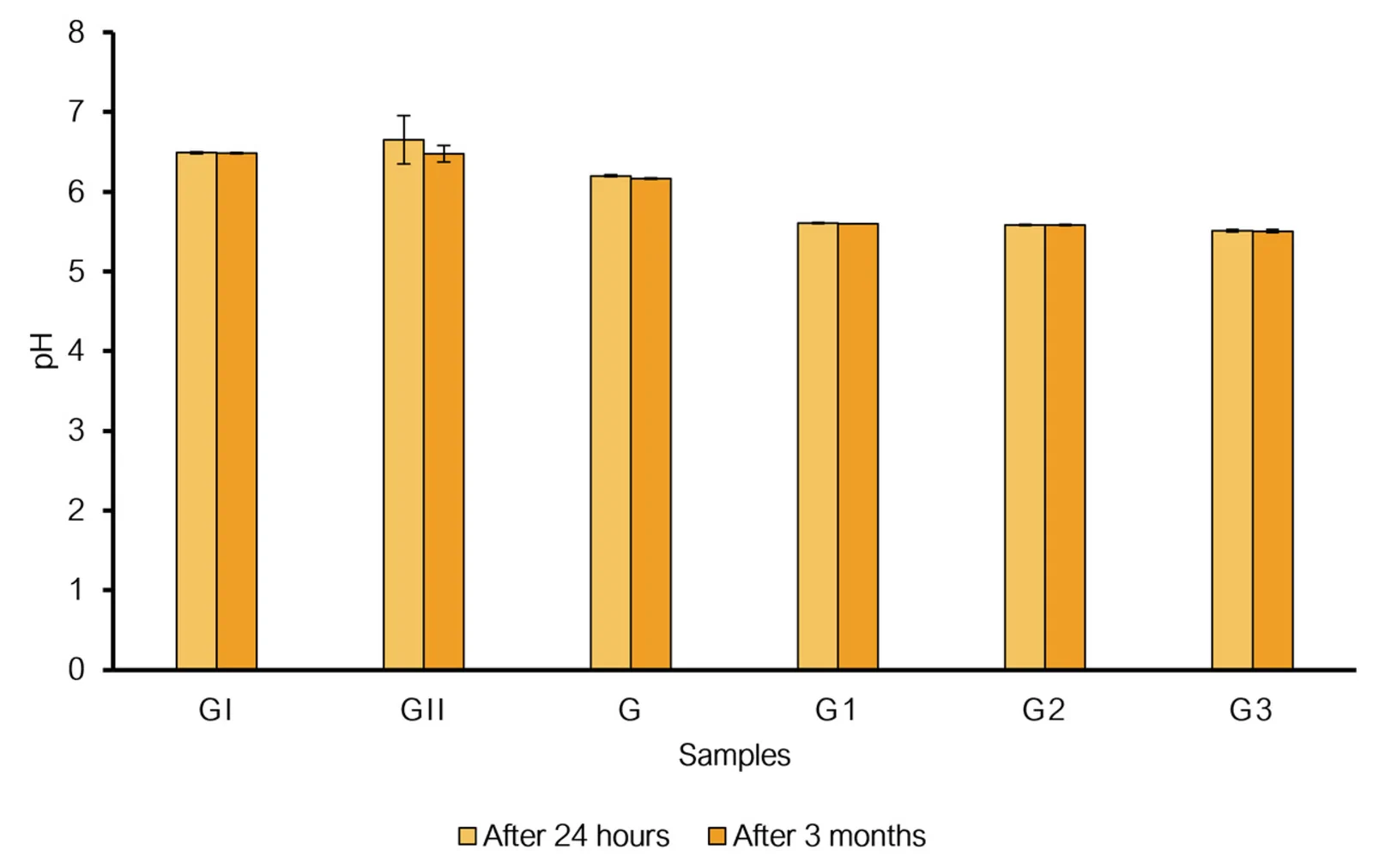
The pH of the gel samples after 24 h and 3 months of storage. Data are presented as mean ± SD (*n* = 5); error bars represent standard deviation.

The pH values of the tested formulations remained generally stable during the 3-month storage period, with only minor changes compared with those measured after 24 h (Figure 7). The blank formulations GI, GII and G showed pH values of approximately 6.1–6.6, whereas incorporation of the *J. regia* extract decreased the pH to approximately 5.5–5.6. Increasing the extract concentration caused only a slight further decrease, with G3 showing the lowest pH value. Only minor changes in pH were observed after 3 months of storage.

After incorporation of the extract into the gel formulations, four phenolic compounds were quantified: ellagic acid pentoside, ellagic acid, quercitrin, and caffeoyl hexose-deoxyhexoside (Table 4). The total quantified content of these compounds increased with increasing extract concentration in the gel, from 10.95 µg/mL in the G1 formulation to 19.17 µg/mL in the G2 formulation and 27.98 µg/mL in the G3 formulation. Ellagic acid was the predominant compound in all gel formulations, reaching 7.49, 11.51, and 17.01 µg/mL in the G1, G2 and G3 formulations, respectively. Quercitrin was also detected in all formulations, with concentrations increasing from 2.96 to 5.63 µg/mL. Ellagic acid pentoside was not detected in the lowest-concentration gel but was quantified in the 0.138% (G2) and 0.184% (G3) formulations at 3.46 and 4.56 µg/mL, respectively. Caffeoyl hexose-deoxyhexoside was present at the lowest concentrations in all gel samples, ranging from 0.497 to 0.783 µg/mL.

Several major ellagic acid-related and flavonoid constituents of the lyophilised extract remained quantifiable in the gel formulations. However, pedunculagin/casuarinin isomers quantified in the lyophilised extract were not quantified in the gel samples. Their non-quantification may be related to the substantially lower concentrations after incorporation of the extract into the gels, reduced recovery from the hydrophilic polymer matrix, matrix-related analytical interference, and/or possible transformation during processing. Since the present study was not designed to distinguish between these mechanisms, the compounds are reported as not quantified rather than absent, and no specific degradation pathway or analytical recovery limitation can be concluded from the present data. The apparent recovery of phenolic compounds varied among the analysed compounds and formulations. Ellagic acid showed the highest apparent recovery, ranging from 106.7% in G1 to 121.2% in G3. Quercitrin recovery ranged from 48.7% to 60.3%, whereas caffeoyl hexose-deoxyhexoside showed recoveries of 40.9–52.5%. Ellagic acid pentoside showed comparable recovery in G2 and G3 (49.6% and 49.0%, respectively) but was not quantifiable in G1. The pedunculagin/casuarin isomers were not quantifiable in any of the gel formulations, and their recovery could therefore not be determined.

Further dedicated stability and recovery studies are needed to clarify the relative contribution of processing-related transformation and matrix effects.

### 3.4. 3D Printing and Physicomechanical Characterization of Films

**Figure 8:**
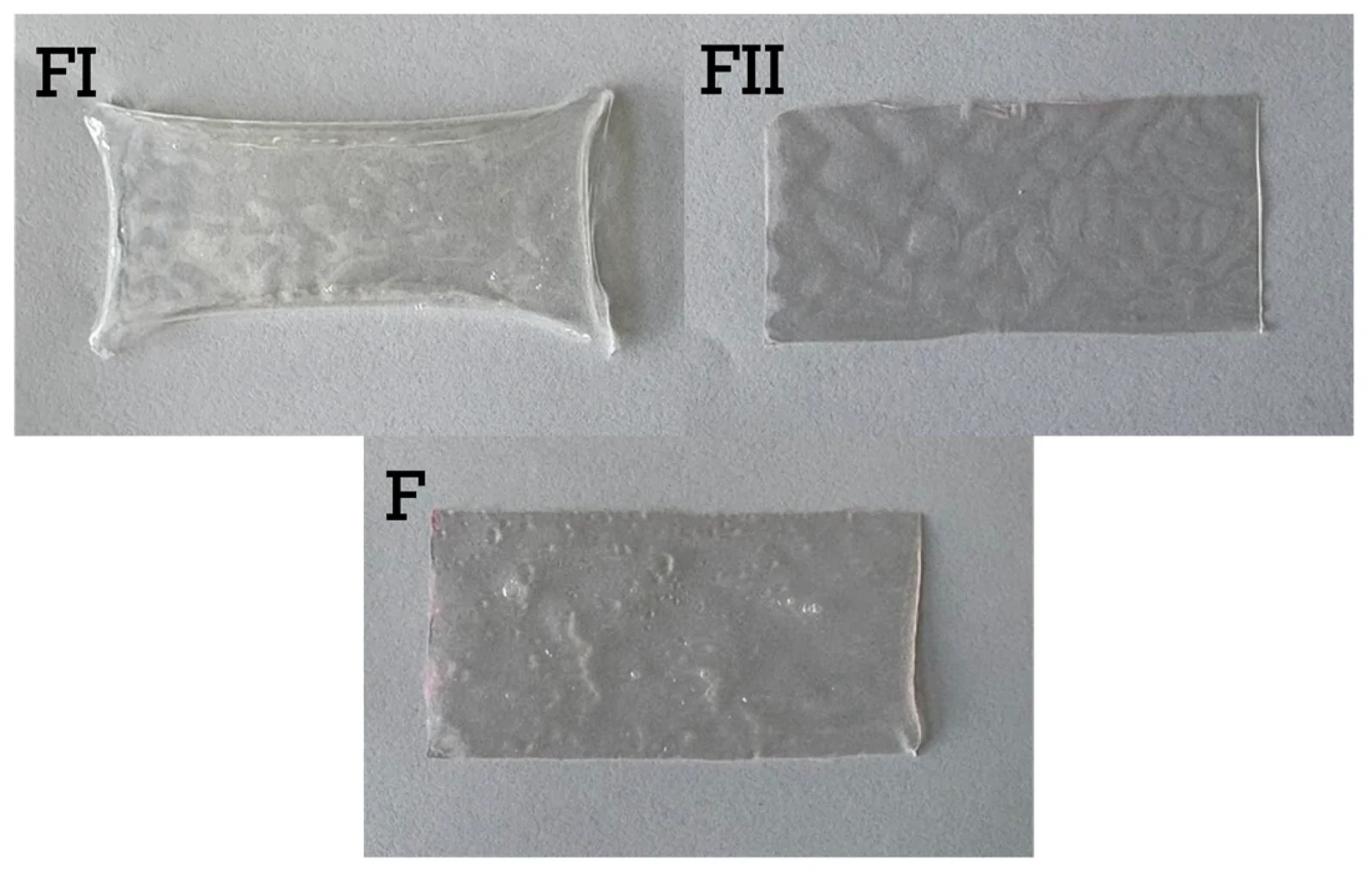
Control film samples prepared by solvent casting in moulds.

During the initial formulation development stage, several polymeric film compositions were evaluated to determine their suitability for semi-solid extrusion 3D printing (Figure 8).

**Figure 9:**
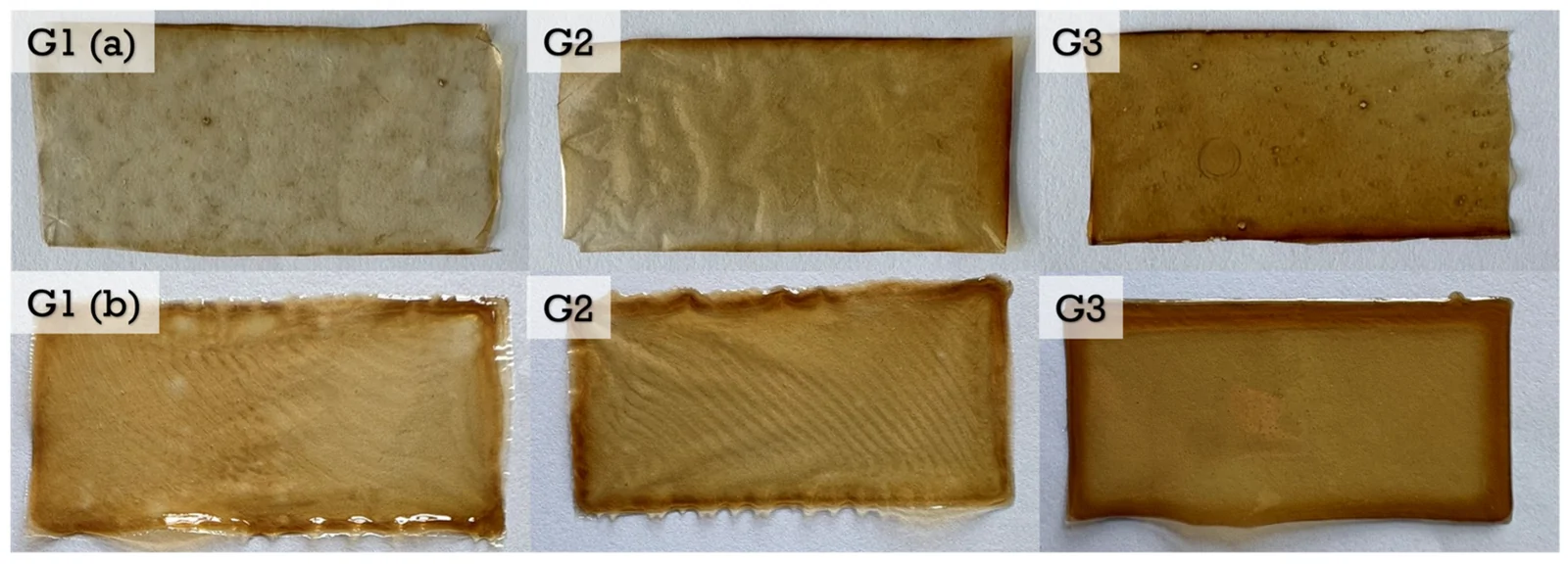
Films prepared from the gel formulations: (**a**) films produced by conventional mould casting and (**b**) films produced by 3D printing.

Figure 8 shows preliminary solvent-cast films prepared during formulation screening. After selecting formulation G (Section 3.3), films were prepared by conventional solvent casting and SSE 3D printing, both with and without lyophilised *J. regia* extract. After selecting formulation G (Section 3.3), films with and without lyophilised *J. regia* extract were prepared by conventional solvent casting and SSE 3D printing and evaluated for their physicomechanical properties (Figure 9).

**Figure 10:**
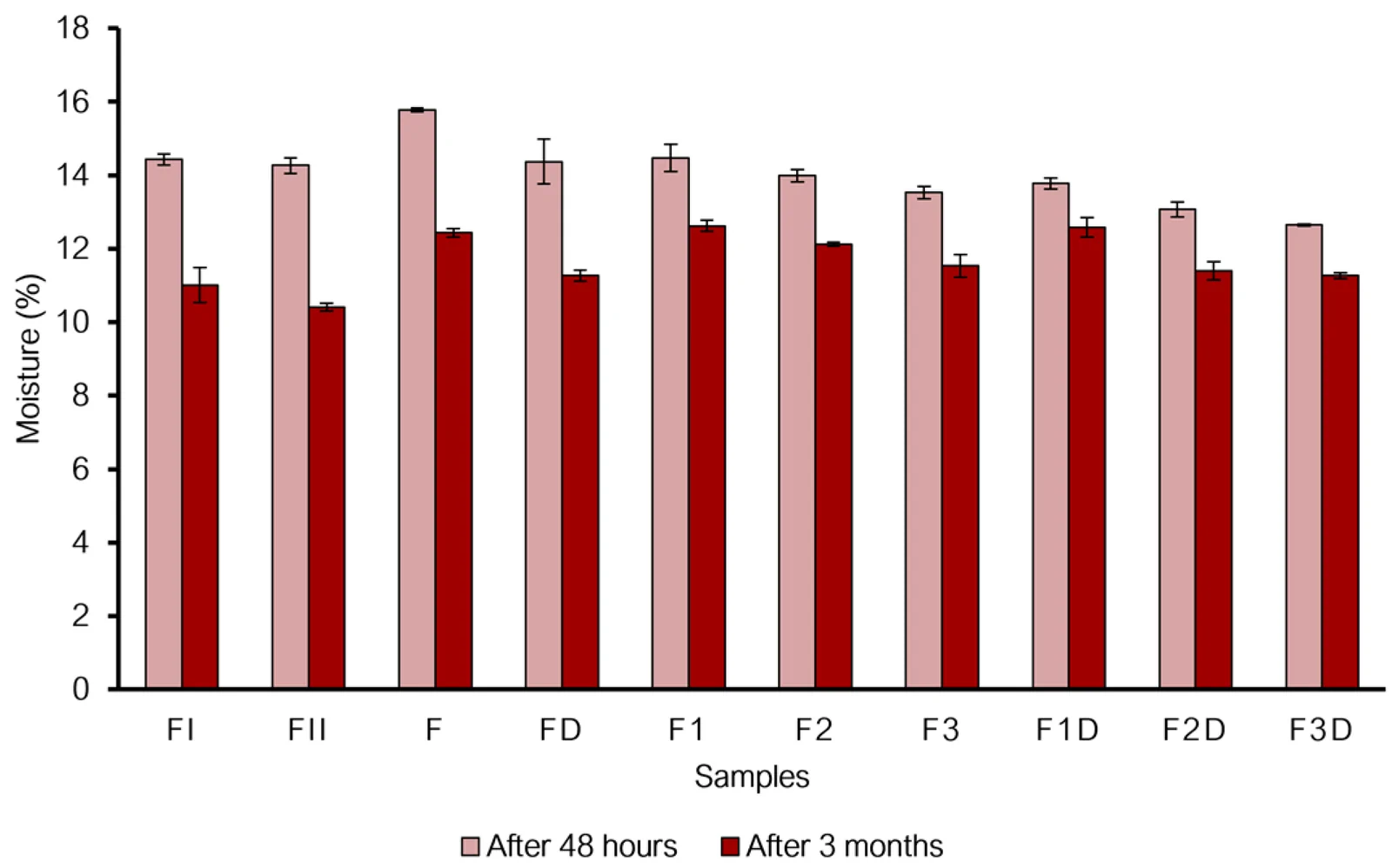
Moisture content of solvent-cast and 3D-printed films after 48 h and 3 months of storage.

The moisture content of the films is presented in Figure 10. The results showed that the manufacturing method and extract incorporation influenced the water content of the prepared films. In general, the 3D-printed films demonstrated lower moisture content than the corresponding cast films. This may be related to the additional thermal exposure during the printing process, as well as the smaller and more uniform deposited layers, which could promote water evaporation during film formation.

The cast blank formulation F showed the highest moisture content after 48 h. In comparison, the 3D-printed films had slightly lower moisture values, which may be explained by additional solvent evaporation during the printing process and by the layer-by-layer structure of the printed samples. After 3 months of storage, moisture content decreased in all formulations, most likely due to evaporation of residual water and solvent. Nevertheless, the cast films generally retained more moisture than the 3D-printed films. The incorporation of lyophilised *Juglans regia* extract also affected the moisture profile of the films. Extract-loaded samples showed differences in moisture content compared with the blank formulations, suggesting that the phenolic-rich dry extract may influence water retention within the polymeric matrix. This effect may be linked to interactions between extract constituents and the hydrophilic polymers used in the formulation. The results indicated that both the preparation method and the presence of the extract contributed to variations in film hydration, which may further influence the mechanical behaviour, flexibility, and stability of the final films.

**Figure 11:**
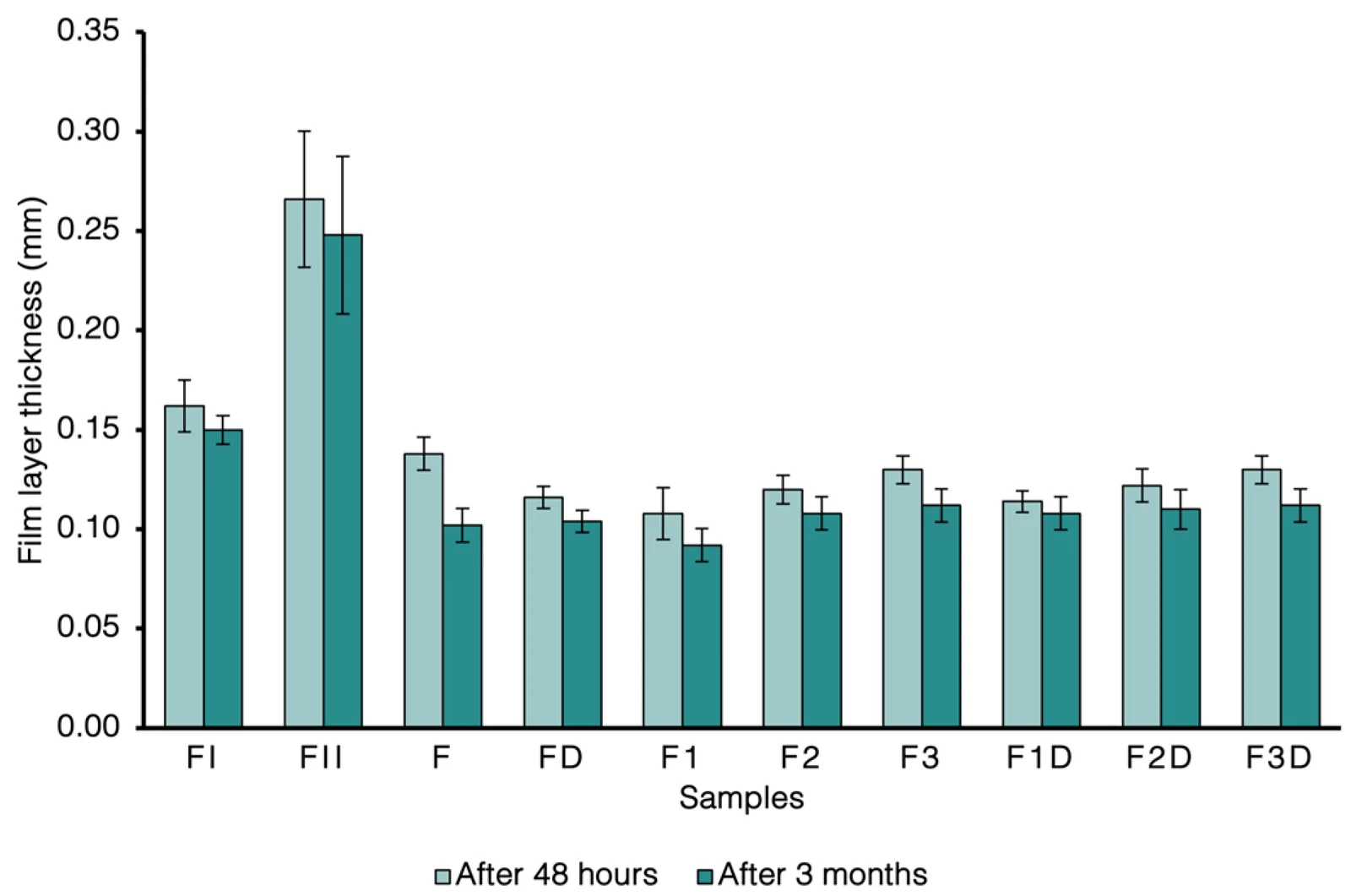
Thickness of solvent-cast and 3D-printed films after 48 h and 3 months of storage.

Film thickness varied with formulation and manufacturing method (Figure 11). Among the initially evaluated formulations, FII produced the thickest films, approximately 0.27 mm after 48 h, followed by FI at approximately 0.16 mm. In comparison, films prepared from the selected formulation F were thinner, at approximately 0.14 mm after 48 h. The corresponding 3D-printed control film (FD) showed a thickness of approximately 0.12 mm.

Within the final extract-containing formulations, film thickness remained within a relatively narrow range. After 48 h, solvent-cast films F1, F2, and F3 had thicknesses of approximately 0.11, 0.12, and 0.13 mm, respectively. A similar pattern was observed for the corresponding 3D-printed films F1D, F2D, and F3D, with thicknesses of approximately 0.11–0.13 mm. Film thickness increased slightly with increasing extract concentration; however, differences between the solvent-cast and 3D-printed films were generally small.

After 3 months of storage, all investigated films showed a reduction in thickness. The decrease was more pronounced for some of the initially developed formulations, particularly F and FII, whereas the extract-containing films showed smaller numerical changes. After storage, both solvent-cast and 3D-printed extract-containing films remained within a similar thickness range of approximately 0.09–0.11 mm. Overall, the 3D-printed films showed thickness values comparable to the corresponding solvent-cast films, indicating that the printing process produced films with dimensions similar to those obtained by conventional casting.

Table 5 presents the structural–mechanical properties of the investigated films. Among the initially screened formulations, FI exhibited the highest burst strength (36.84 ± 4.52 N), followed by FII (21.83 ± 3.99 N), while the selected formulation F showed a lower value of 14.67 ± 2.45 N. A similar pattern was observed for tensile breaking strength per unit width, which was highest for FI (1.678 ± 0.148 N/mm), followed by FII (0.933 ± 0.076 N/mm) and F (0.785 ± 0.121 N/mm).

The 3D-printed films showed numerically lower mechanical strength values than the corresponding solvent-cast films. The 3D-printed control film FD showed a numerically lower burst strength (10.05 ± 1.43 N) than the corresponding solvent-cast film F (14.67 ± 2.45 N). Likewise, tensile breaking strength per unit width was 0.687 ± 0.049 N/mm for FD compared with 0.785 ± 0.121 N/mm for F.

In the extract-containing solvent-cast films, burst strength decreased gradually with increasing *Juglans regia* extract concentration, from 13.78 ± 2.00 N for F1 to 12.84 ± 2.11 N for F2 and 11.34 ± 2.42 N for F3. The same tendency was observed for the corresponding 3D-printed films, with values of 9.96 ± 1.51, 9.42 ± 1.83, and 9.13 ± 1.38 N for F1D, F2D, and F3D, respectively.

Tensile breaking strength varied less among the solvent-cast extract-containing films, with values of 0.701 ± 0.089 N/mm for F1, 0.678 ± 0.105 N/mm for F2, and 0.681 ± 0.098 N/mm for F3. The corresponding 3D-printed films showed values of 0.596 ± 0.056, 0.587 ± 0.041, and 0.556 ± 0.063 N/mm, respectively. Overall, the 3D-printed films showed numerically lower burst strength and tensile breaking strength per unit width than their corresponding solvent-cast formulations. Increasing extract concentration was accompanied by a gradual numerical decrease in burst strength, whereas changes in tensile breaking strength were less pronounced.

Statistical analysis showed significant overall differences among the samples for both film burst strength (F(9,40) = 55.25, *p* < 0.0001) and breaking force per unit width (F(9,40) = 66.72, *p* < 0.0001). FI showed significantly higher values than all other samples. However, we found no significant differences between the corresponding solvent-cast and 3D-printed pairs F/FD, F1/F1D, F2/F2D, and F3/F3D (*p* > 0.05).

The adhesion properties of the investigated films are presented in Table 6. Hydration increased the mean adhesiveness and work of adhesion of all formulations, although the statistical significance of this effect differed between the two parameters. Among the initially tested films, FI showed an adhesiveness of 0.36 ± 0.06 N in the dry state, which increased to 5.17 ± 0.55 N after wetting. Its work of adhesion similarly increased from 0.04 ± 0.01 to 0.67 ± 0.05 N·s. FII showed the lowest dry-state adhesiveness, 0.11 ± 0.02 N, which increased to 1.33 ± 0.11 N following hydration.

For the selected formulation, the solvent-cast control film F showed an adhesiveness of 0.96 ± 0.03 N in the dry state and 2.54 ± 0.19 N after wetting. The corresponding 3D-printed control film FD exhibited values of 1.03 ± 0.11 and 3.84 ± 0.44 N, respectively. The work of adhesion increased from 0.12 ± 0.02 to 0.24 ± 0.02 N·s for F and from 0.09 ± 0.02 to 0.36 ± 0.11 N·s for FD after hydration.

Among the extract-containing solvent-cast films, dry-state adhesiveness increased from 1.08 ± 0.10 N for F1 to 1.40 ± 0.13 N for F3. The corresponding 3D-printed films showed slightly higher values, ranging from 1.21 ± 0.07 N for F1D to 1.72 ± 0.13 N for F3D. After wetting, adhesiveness ranged from 2.58 ± 0.21 to 2.87 ± 0.16 N for the solvent-cast films and from 3.60 ± 0.26 to 3.80 ± 0.41 N for the 3D-printed films.

A similar tendency was observed for the work of adhesion. In the dry state, values ranged from 0.13 ± 0.01 to 0.17 ± 0.05 N·s for the solvent-cast extract-containing films and from 0.10 ± 0.02 to 0.17 ± 0.08 N·s for their 3D-printed counterparts. After hydration, the extract-containing formulations showed the highest work of adhesion for F3D (0.67 ± 0.22 N·s), followed by F3 (0.55 ± 0.20 N·s). Overall, hydration increased probe-film adhesiveness under the test conditions, while the 3D-printed extract-containing films generally exhibited higher adhesiveness after wetting than the corresponding solvent-cast films.

Statistical analysis showed significant overall differences among the samples for both adhesiveness (F(19,80) = 171.88, *p* < 0.0001) and work of adhesion (F(19,80) = 21.03, *p* < 0.0001). Tukey’s multiple-comparisons test showed that hydration significantly increased adhesiveness for all formulations (*p* < 0.05). For work of adhesion, hydration significantly increased values for FI, FII, FD, F3, F2D, and F3D, whereas changes for F, F1, F2, and F1D were not statistically significant. Hydrated 3D-printed films showed significantly higher adhesiveness than their corresponding solvent-cast films, while no significant differences in work of adhesion were found between the corresponding manufacturing methods.

## 4. Discussion

The protective effect of *Juglans regia* extract observed in fibroblasts may be associated with its phenolic composition. The extract contained six quantified phenolic constituents, including two pedunculagin/casuarin isomers, ellagic acid pentoside, ellagic acid, quercitrin, and caffeoyl hexose-deoxyhexoside. This profile is consistent with our previous phytochemical investigations of *J. regia* and other reports identifying ellagitannins, ellagic acid derivatives, flavonoids, and caffeoyl derivatives as characteristic walnut constituents [5,37]. Among these compounds, ellagic acid is particularly relevant because Baek et al. showed that it reduced intracellular ROS and restored antioxidant defences in human dermal fibroblasts exposed to oxidative stress [38]. Quercitrin has likewise been reported to reduce ROS generation, oxidative damage, and cell death in a skin-related oxidative-stress model [39]. The detected pedunculagin/casuarin isomers may also contribute to the antioxidant activity of the extract, as walnut ellagitannins, including pedunculagin-related compounds, have demonstrated strong radical-scavenging and superoxide dismutase-like activity [37,40]. Caffeoyl derivatives have also been reported in *J. regia* tissues, although direct evidence linking caffeoyl hexose-deoxyhexoside specifically to fibroblast protection remains limited [5,41,42,43].

Overall, the observed cytoprotective effect is likely associated with the combined activity of the multicomponent phenolic profile rather than a single constituent. Interactions among phenolic compounds may influence the biological response [44,45]. However, the contribution and mechanism of individual constituents cannot be established from the present experiments. Studies using isolated compounds, defined combinations, or fractionated extracts would be required to clarify their individual roles [46]. Importantly, the cytoprotective effect demonstrated in this study relates to the free *J. regia* extract. Extract release from the polymeric matrices was not assessed, formulation loadings cannot be directly related to the free-extract concentrations used in the fibroblast experiments.

The development of the film began with the selection of an appropriate gel formulation to be suitable for topical application while also possessing the rheological and processing properties required for semi-solid extrusion (SSE) 3D printing. It required a balance between suitable consistency, homogeneity, and practical extrudability. The rheological properties of the formulation directly affect material flow through the nozzle, deposition accuracy, and the ability of the deposited material to retain its shape after extrusion [28,47]. The initially investigated formulations GI and GII showed higher consistency, whereas GIII was less thick, homogeneous and contained no visible dispersed particles that could interfere with extrusion. For this reason, GIII was selected for further studies and subsequently designated as G. A limitation of the present study is the absence of shear-rate-dependent flow curves, oscillatory measurements, and thixotropic recovery testing. Therefore, parameters such as yield stress, shear-thinning index, G′, G″, tanδ, and structural recovery could not be determined. The suitability of formulation G for SSE printing was established empirically under the selected processing conditions rather than through comprehensive rheological characterisation.

Incorporation of lyophilised *Juglans regia* extract at 0.092–0.184% (*w*/*w*) did not significantly alter the apparent viscosity of the selected gel formulation, and only minor changes in apparent viscosity and pH were observed after 3 months of storage. The extract-containing gels remained mildly acidic (approximately pH 5.5–5.6). These findings indicate that the measured pH and apparent viscosity remained relatively stable during the 3-month storage period and support further evaluation of the formulations for topical application and SSE 3D printing. Similar feasibility has been reported by Koshovyi et al., who successfully incorporated eucalypt extract into a PEO-based SSE-printable gel after optimisation of formulation and printing conditions [30].

The concentration-dependent increase in quantified phenolic compounds after incorporation of the *J. regia* extract indicates successful transfer of its major constituents into the gel matrix, with ellagic acid remaining the predominant detected compound. Ellagic acid pentoside was not quantified in G1, possibly because its concentration was below the analytical detection or quantification threshold [48]. Ellagic acid and quercitrin have similarly been used as marker compounds in botanical topical formulations [49,50]. Overall, the gel formulations retained several major phenolic constituents of the extract, although apparent recovery varied among compounds. Quercitrin and caffeoyl hexose-deoxyhexoside showed moderate recovery, ellagic acid pentoside was quantifiable only in G2 and G3, and ellagic acid showed apparent recovery slightly above 100%, likely due to analytical or matrix-related variability.

The selected G formulation was processed by both solvent casting and SSE 3D printing under comparable formulation conditions (formulation mass (6.9 g) and target dimensions 40 × 80 mm). Unlike solvent casting, SSE relies on sequential layer deposition; therefore, a 180 s interlayer pause was introduced empirically to promote partial solvent evaporation and stabilisation of the deposited structure [51,52]. The similar final thicknesses of cast and printed films indicate that SSE produced macroscopically comparable films, although differences in internal structure, solvent removal, and polymer organisation may still influence moisture retention and mechanical behaviour [36,53,54]. Because the formulations contain hydrophilic polymers and PEG 400, changes in thickness should be interpreted together with residual moisture, as PEG 400 can affect water transport and mechanical properties [55,56]. Thus, combined evaluation of thickness and moisture content provides a more informative assessment of physical changes during storage than either parameter alone. Although differences in film formation may influence internal structure and consequently bioactive release, porosity and microstructure were not directly evaluated in the present study. Comparative in vitro release and permeation studies using diffusion-cell methods are planned to determine whether solvent-cast and SSE 3D-printed films differ in the release of marker phenolics such as ellagic acid and quercitrin.

The mechanical results indicate that formulation selection could not be based solely on film strength. Although FI and FII showed higher mechanical strength, the corresponding GI and GII gels were less suitable for SSE because of their higher consistency. This illustrates the balance required between sufficient structural integrity and adequate flow through the nozzle, as excessive viscosity or viscoelastic resistance can impair extrusion and promote clogging [57,58]. Similar formulation–process relationships have been reported by Seoane-Viaño et al. [58] and Koshovyi et al. [30]. Accordingly, formulation G/F represented a more appropriate compromise between printability and final film integrity. This is also consistent with reports showing that polymer type, concentration, and plasticiser composition strongly influence the mechanical properties of polymeric films [23].

The manufacturing method also influenced film mechanical behaviour, with 3D-printed films generally showing numerically lower burst strength and breaking force per unit width than their solvent-cast counterparts. This may be related to differences in film formation, as solvent casting produces a continuous matrix whereas SSE printing creates sequentially deposited layers with interlayer interfaces and a different drying history. Similar manufacturing-dependent effects have been reported for PVA-based films [53]. However, lower strength is not an inherent limitation of SSE, since comparable mechanical properties can be achieved after optimisation of formulation and printing conditions [59]. Increasing *J. regia* extract loading was accompanied by a gradual numerical decrease in burst strength, while changes in breaking force per unit width were less pronounced. This may reflect extract-induced changes in polymer-matrix interactions. Similar effects have been reported for plant extract-containing PVA systems, although their magnitude depends on both extract and polymer composition [60]. Therefore, the observed mechanical changes should be considered formulation-specific.

Hydration strongly increased probe–film adhesiveness in all formulations, consistent with water-induced swelling and greater surface deformability of hydrophilic polymer matrices [23,53,61]. Hydrated 3D-printed films generally showed higher adhesiveness than their solvent-cast counterparts, possibly reflecting surface characteristics created by layer-by-layer deposition. However, this mechanism was not directly investigated, therefore these findings indicate that mechanical strength and adhesion should be considered complementary rather than directly related, and differences between manufacturing methods should be interpreted in the context of both film structure and hydration state.

An important limitation of the present study is that the cytoprotective effect was demonstrated only for the free lyophilised *J. regia* extract. Although major phenolic compounds were detected after incorporation of the extract into the gel formulations, their presence alone does not demonstrate that the cytoprotective activity of the free extract was preserved after formulation. The final gels and films were not evaluated in the fibroblast model, and release of the extract constituents from the polymeric matrices was not assessed. Therefore, the biological activity observed for the free extract cannot be directly extrapolated to the developed formulations. Future studies should evaluate extract release and the biological activity of the final gel and film formulations.

Overall, the results demonstrate the importance of considering the complete formulation-to-product pathway rather than selecting a formulation on the basis of a single mechanical parameter. Although GI and GII produced mechanically stronger films, their higher consistency limited their suitability for SSE printing. In contrast, formulation G provided appropriate extrusion characteristics, remained physically stable after incorporation of up to 0.184% (*w*/*w*) lyophilised *J. regia* extract, and could be successfully processed by both solvent casting and SSE 3D printing. Importantly, the developed formulation may therefore be considered a versatile platform, as the extract-containing gel can potentially be used directly as a topical semisolid preparation or further transformed into a film when a solid, more localised application is preferred. The 3D-printed films showed thicknesses comparable to the corresponding solvent-cast films and retained measurable mechanical strength, although their burst and tensile breaking strengths were numerically lower, while hydration generally increased their adhesiveness.

## 5. Conclusions

The present study demonstrated a cytoprotective effect of free lyophilised *Juglans regia* L. septum extract, containing ellagic acid and its derivatives, quercitrin, caffeoyl derivatives, and hydrolysable tannin-related compounds, in fibroblasts exposed to H_2_O_2_-induced oxidative stress. The greatest preservation of viable fibroblasts was observed at extract concentrations of 75 and 100 µg/mL. Separately, the extract was successfully incorporated into polymeric gels that could be processed into films by conventional casting and SSE 3D printing. Increasing the extract concentration in the gels increased the quantified phenolic content from 10.95 µg/mL in G1 to 27.98 µg/mL in G3, and produced formulation-dependent variations in physicochemical and mechanical properties. However, biological activity was evaluated only for the free extract, and the final gel and film formulations were not tested in the fibroblast model. Therefore, the present study does not establish whether the cytoprotective activity of the extract was preserved after incorporation into the formulations.

The pH of the developed gels remained suitable for topical skin application with extract-containing formulations showing values of approximately 5.5–5.6. Formulation G was selected based on its apparent viscosity, homogeneous appearance, qualitative extrusion behaviour, and the characteristics of the resulting films, rather than solely on film mechanical strength. SSE 3D printing successfully produced multilayer films. The corresponding 3D-printed films generally showed numerically lower burst strength and tensile breaking strength than the solvent-cast films. The corresponding 3D-printed films generally showed numerically lower burst strength and breaking force per unit width than the solvent-cast films, although the corresponding pairwise differences were not statistically significant. These pairwise mechanical differences were not statistically significant. Hydration significantly increased probe–film adhesiveness across all formulations, whereas adhesion increased significantly only for selected formulations. Film moisture content decreased in all formulations during the 3-month storage period, indicating changes in the evaluated physical properties rather than demonstrating overall formulation stability.

Overall, extract concentration, formulation composition, and manufacturing method influenced the physicochemical and mechanical properties of the developed films. Further studies should address long-term stability, release and permeation of marker phenolics, and biological activity of the final formulations.

## Figures and Tables

**Table 1:** Composition of initial control gel formulations, % *w*/*w*.

Component	Formulation GI, % *w*/*w*	Formulation GII, % *w*/*w*	Formulation GIII (G), % *w*/*w*
PVA	4.656	-	1.631
CMC	1.171	-	0.408
HEC	0.579	1.145	0.203
MC	-	1.294	-
PEG 400	2.328	4.871	1.304
Propylene glycol	2.328	1.610	1.599
Ethanol	20.183	11.924	16.697
Sodium hyaluronate	0.135	0.083	0.041
D-panthenol	1.346	0.830	0.816
Purified water	67.277	78.242	77.301

**Table 2:** Composition of control and *Juglans regia* L. extract-loaded gel formulations, % *w*/*w*.

Component	Control (G), % *w*/*w*	G1, % *w*/*w*	G2, % *w*/*w*	G3, % *w*/*w*
PVA	1.631	1.631	1.631	1.631
CMC	0.408	0.408	0.408	0.408
HEC	0.203	0.203	0.203	0.203
PEG 400	1.304	1.304	1.304	1.304
Propylene glycol	1.599	1.599	1.599	1.599
Ethanol	16.697	16.697	16.697	16.697
Sodium hyaluronate	0.041	0.041	0.041	0.041
D-panthenol	0.816	0.816	0.816	0.816
Purified water	77.301	77.208	77.162	77.116
Lyophilised *Juglans regia* L. extract	0.000	0.092	0.138	0.184

**Table 3:** Quantified phenolic compounds in the lyophilised *Juglans regia* L. extract.

Determined Compounds	Concentration, μg/mL
Pedunculagin/casuarin isomer 1	13.891
Pedunculagin/casuarin isomer 2	15.098
Ellagic acid pentoside	24.144
Ellagic acid	36.453
Quercitrin	25.501
Caffeoyl hexose-deoxyhexoside	4.95

**Table 4:** Quantified phenolic compounds in extract-loaded gel formulations (µg/mL).

Determined Compound	G1, µg/mL	G2, µg/mL	G3, µg/mL
Ellagic acid pentoside	NQ	3.46	4.56
Ellagic acid	7.49	11.51	17.01
Quercitrin	2.96	3.59	5.63
Caffeoyl hexose-deoxyhexoside	0.50	0.61	0.78
Pedunculagin/casuarin isomer 1	NQ	NQ	NQ
Pedunculagin/casuarin isomer 2	NQ	NQ	NQ

NQ—non quantified.

**Table 5:** Structural-mechanical properties (*n* = 5).

Sample *	Film Burst Strength, N **	Breaking Force per Unit Width (N/mm) **
FI	36.84 ± 4.52 ^a^	1.678 ± 0.148 ^a^
FII	21.83 ± 3.99 ^b^	0.933 ± 0.076 ^b^
F	14.67 ± 2.45 ^c^	0.785 ± 0.121 ^bd^
FD	10.05 ± 1.43 ^cd^	0.687 ± 0.049 ^cd^
F1	13.78 ± 2.00 ^cd^	0.701 ± 0.089 ^cd^
F2	12.84 ± 2.11 ^cd^	0.678 ± 0.105 ^cd^
F3	11.34 ± 2.42 ^cd^	0.681 ± 0.098 ^cd^
F1D	9.96 ± 1.51 ^cd^	0.596 ± 0.056 ^cd^
F2D	9.42 ± 1.83 ^cd^	0.587 ± 0.041 ^c^
F3D	9.13 ± 1.38 ^d^	0.556 ± 0.063 ^c^

* F, solvent-cast control film; FD, 3D-printed control film; F1–F3, solvent-cast extract-containing films; F1D–F3D, corresponding 3D-printed films. ** Statistical differences were evaluated separately for film burst strength and breaking force per unit width using one-way analysis of variance (ANOVA) followed by Tukey’s multiple-comparisons post hoc test. Lowercase superscript letters denote statistical groupings within each column. Values with no superscript letter in common differ significantly (*p* < 0.05), whereas values sharing at least one letter do not differ significantly (*p* > 0.05).

**Table 6:** Adhesiveness and work of adhesion of dry and hydrated films (*n* = 5).

Sample *	Probe-Film Adhesiveness, N **	Work of Adhesion N·s **
FI	0.36 ± 0.06 ^g^	0.04 ± 0.01 ^gh^
FI + W	5.17 ± 0.55 ^a^	0.67 ± 0.05 ^a^
FII	0.11 ± 0.02 ^g^	0.01 ± 0.00 ^h^
FII + W	1.33 ± 0.11 ^def^	0.27 ± 0.04 ^cdef^
F	0.96 ± 0.03 ^f^	0.12 ± 0.02 ^efgh^
F + W	2.54 ± 0.19 ^c^	0.24 ± 0.02 ^cdefg^
FD	1.03 ± 0.11 ^ef^	0.09 ± 0.02 ^fgh^
FD + W	3.84 ± 0.44 ^b^	0.36 ± 0.11 ^bcd^
F1	1.08 ± 0.10 ^ef^	0.13 ± 0.01 ^efgh^
F1+ W	2.58 ± 0.21 ^c^	0.27 ± 0.08 ^cdef^
F2	1.32 ± 0.08 ^def^	0.16 ± 0.06 ^defgh^
F2 + W	2.66 ± 0.22 ^c^	0.31 ± 0.14 ^cde^
F3	1.40 ± 0.13 ^def^	0.17 ± 0.05 ^defgh^
F3 + W	2.87 ± 0.16 ^c^	0.55 ± 0.20 ^ab^
F1D	1.21 ± 0.07 ^def^	0.10 ± 0.02 ^efgh^
F1D + W	3.6 ± 0.26 ^b^	0.29 ± 0.07 ^cdef^
F2D	1.54 ± 0.09 ^de^	0.12 ± 0.03 ^efgh^
F2D + W	3.77 ± 0.31 ^b^	0.41 ± 0.16 ^bc^
F3D	1.72 ± 0.13 ^d^	0.17 ± 0.08 ^defgh^
F3D + W	3.80 ± 0.41 ^b^	0.67 ± 0.22 ^a^

* F, solvent-cast control film; FD, 3D-printed control film; F1–F3, solvent-cast extract-containing films; F1D–F3D, corresponding 3D-printed films. +W indicates samples hydrated with water prior to adhesion testing.** Statistical differences were evaluated separately for adhesiveness and work of adhesion using one-way analysis of variance (ANOVA) followed by Tukey’s multiple-comparisons post hoc test. Lowercase superscript letters (a–h) denote statistical groupings within each column. Values with no superscript letter in common differ significantly *(p* < 0.05), whereas values sharing at least one letter do not differ significantly (*p* > 0.05). Statistical groupings were determined independently for adhesiveness and work of adhesion.

## Data Availability

Data are contained within the article.

## Abbreviations

The following abbreviations are used in this manuscript:

ATCC	American Type Culture Collection
CMC	Sodium carboxymethyl cellulose
DMEM	Dulbecco’s Modified Eagle Medium
EDTA	Ethylenediaminetetraacetic acid
FBS	Fetal bovine serum
HEC	Hydroxyethyl cellulose
HPLC-PDA	High-performance liquid chromatography with photodiode array detection
HPMC	Hydroxypropyl methylcellulose
MC	Methylcellulose
PEG 400	Polyethylene glycol 400
PEO	Polyethylene oxide
PVA	Poly(vinyl alcohol)
RRID	Research Resource Identifier
SSE	Semi-solid extrusion
